# Pure Uterine Lipoma with a Combined Mucinous Cystic and Brenner Ovarian Tumor in a Postmenopausal Woman: A Case Report and Review of the Literature

**DOI:** 10.1155/2021/5512951

**Published:** 2021-03-29

**Authors:** Toshimitsu Tohya, Mariko Tominaga, Tomoko Honda

**Affiliations:** Department of Obstetrics and Gynecology, Kumamoto Rosai Hospital, Kumamoto 866-8533, Japan

## Abstract

Lipomatous tumors are rare among uterine mesenchymal tumors. Due to their rarity, information on the clinical development and histological origin are lacking. We report a pure uterine lipoma and present a review of the relevant literature. We encountered a 72-year-old postmenopausal woman who was referred to our hospital. Computed tomography (CT) and magnetic resonance imaging (MRI) examinations revealed degenerative uterine tumors and a left ovarian cystic tumor with a solid portion. Total hysterectomy and bilateral salpingo-oophorectomy were performed, and postoperative histological examination revealed a uterine lipoma and adenomyosis. The cystic portion was a mucinous cystadenoma, and the solid portion was classified as a Brenner tumor. The postoperative course was uneventful, and the patient was discharged and remained well until follow-up. Our observations suggest that imaging examinations are sufficient for the diagnosis of uterine lipomatous tumors. To the best of our knowledge, the coexistence of a pure uterine lipoma and a mucinous Brenner ovarian tumor has not been documented in the existing literature. The histogenesis of uterine lipomas remains unclear. However, our results, and those from the existing literature, indicate that the mesenchymal stem cells surrounding the perivascular tissue may be implicated, because lipomas of the skin are reported to originate from these cells.

## 1. Introduction

Mesenchymal tumors of the uterus, especially leiomyomas, are the most common tumors of the female genital tract [1]. However, lipomatous uterine tumors are rare among mesenchymal uterine tumors. The incidence varies from 0.03 to 0.2% [2]. Lipomatous tumors can be divided into two groups, lipoleiomyomas and pure lipomas. Due to the rarity of lipoleiomyomas, information on their clinical development and histological origin is lacking, with a brief mention in the World Health Organization (WHO) classification of tumors in the female reproductive organ (WHO Press, 2014). We report a case of pure uterine lipoma and present a review of the relevant literature.

## 2. Case Presentation

**Figure 1:**
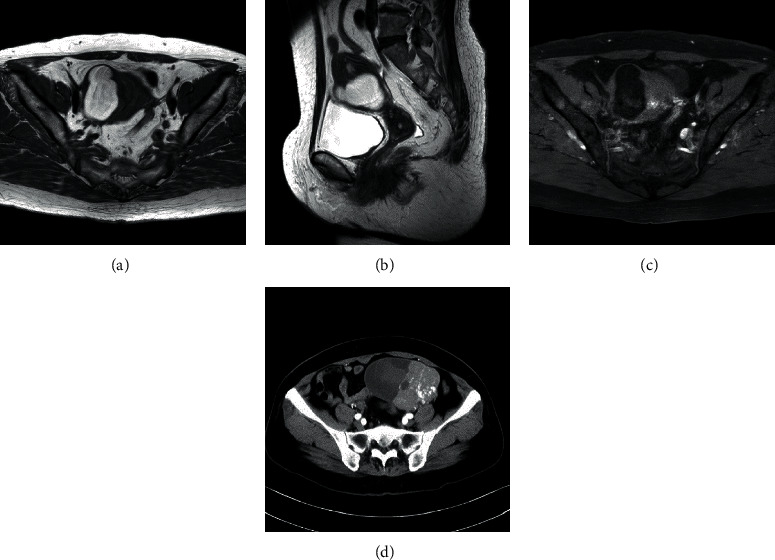
Imaging of the uterine lipoma and the ovarian tumor. The uterine lipoma was examined by MRI: (a) axial T1 weighted imaging (T1WI); (b) sagittal T2 weighted imaging (T2WI); (c) fat suppression T1WI; (d) enhanced CT examination of the left ovarian tumor.

A 72-year-old woman was referred to our hospital for uterine and ovarian tumors. The patient had three prior pregnancies, two of which resulted in live births. She also had chronic hypertension, which was being managed with antihypertensive medication. The patient's family history was unremarkable. The patient had experienced scanty abnormal genital bleeding 3 months prior. Ultrasonography, computed tomography (CT), and magnetic resonance imaging (MRI) examinations (Figure 1) revealed degenerative uterine tumors and a left ovarian tumor containing both cystic and solid portions. The uterine tumors were suspected to be lipomatous because of the intensity of T1- and T2-weighted MRI (Figure 1). She was hospitalized after the diagnosis since the possibility of malignancy in the ovarian tumor could not be ruled out.

Laboratory examinations upon admission, such as complete blood count, blood chemistry, and tumor markers (e.g., carbohydrate antigen 125), were almost normal.

**Figure 2:**
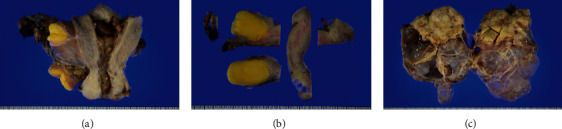
Macroscopic appearance of the resected uterus, left ovarian tumor: (a) macroscopic examination following hysterectomy; (b) macroscopic examination of the uterine lipoma on the cut surface; (c) macroscopic examination of the ovarian tumor following salpingo-oophorectomy.

**Figure 3:**
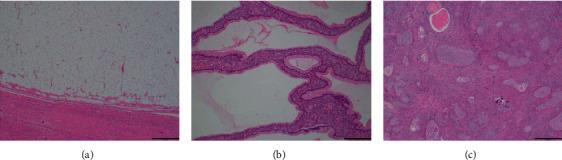
Microscopic findings of the uterine lipoma and the left ovarian tumor: (a) microscopic visualization of fat cells in the uterine lipoma; (b) histological analysis of the mucinous portion of the left ovarian tumor; (c) histological analysis of the solid portion of the left ovarian tumor.

Total hysterectomy and bilateral salpingo-oophorectomy were performed. The macroscopic appearance of the uterine tumor was characteristic of a lipoma (Figure 2). Postoperative histological examination revealed lipomatous uterine tissue and adenomyosis. The solid portion of the left ovarian tumor was classified as a Brenner tumor, and the cystic portion was classified as a mucinous cystadenoma (Figure 3). The postoperative course was uneventful, and the patient was discharged on the 10th postoperative day. The patient remained well as observed at the 18-month follow-up.

## 3. Discussion

Lipomatous uterine tumors have not received significant attention in the gynecologic clinical field due to their rarity and benign nature. However, their histogenesis is interesting. They are frequently associated with preoperative diagnostic confusion. Imaging examinations, such as CT and MRI, are effective for the diagnosis of lipomatous uterine tumors [3] because the lipid component of these tumors is easily detected using these techniques [2]. Histologically, these tumors encompass a spectrum of tumors including pure lipomas, lipoleiomyomas, and lipofibromas based on the relative proportion of fat, smooth muscle, and fibrous tissue present in the tumor [4]. Although the final diagnosis requires histological examination, hysterectomy may be unnecessary in most patients, as these tumors are frequently benign and often occur in older women. Surgical intervention should be preferred only when malignancy is suspected or when other tumors are present as observed in our patient [2]. However, cases of liposarcoma in lipoleiomyoma [5] or leiomyosarcoma in lipoleiomyoma [6] have also been reported. After investigations, such as CT, MRI, and blood chemistry, clinicians may choose not to treat asymptomatic cases, whereas in symptomatic patients, treatment includes hysterectomy.

We reviewed the literature related to lipomatous uterine tumors published between 1990 and 2020. All literature was obtained from a PubMed search, and only articles written in English were considered. Lipomatous tumors typically occurred in postmenopausal women aged 50-70 years, and most patients were asymptomatic [1]. Some of these were coincidently associated with other lesions, including struma ovarii, endometrial carcinoma [7], and cervical carcinoma. To the best of our knowledge, this appears to be the first case report describing a pure uterine lipoma associated with a mucinous cystic and Brenner ovarian tumor. Our PubMed search yielded 69 publications, of which 33 described pure lipomas and 36 described lipoleiomyomas. Pure lipomas were present almost exclusively in the uterus, with only 3 lipomas in the round and broad ligaments. Lipoleiomyoma was more complex, with 5 cases in the broad ligament and retroperitoneum, 2 cases in the peritoneum, 1 case visible from the vena cava to the heart [8], and 1 case with lung metastasis [9]. These cases are summarized in Table 1.

Lipomatous uterine tumors were reported at various sites. However, their histogenesis remained unclear. Because fat tissue is not normally found in the uterus, several theories for the histogenesis of these tumors have been proposed. These theories include misplaced embryonic fat cells, metaplasia of connective tissue or muscle cells into adipocytes, lipocytic differentiation of specific primitive connective tissue cells, proliferation of perivascular fat cells, and fatty infiltration or degeneration of the connective tissue. We hypothesize that lipomatous uterine tumors are derived from mesenchymal stem cells surrounding the perivascular tissue. This hypothesis evolved from research showing that lipomas of the skin can originate from mesenchymal stem cells surrounding the perivascular tissue [10]. Overall, our observations and literature review highlight a novel potential mechanism that could also explain the vascular extension or lung metastasis reported in the literature.

## Figures and Tables

**Table 1:** Variant and associated lesion of uterine lipomatous tumor.

Uterine lipomatous tumor	Ectopic tumor	Associated lesion
Lipoleiomyoma	Broad ligament, 4 papers Abdominal wall, 2 papers Vena cava, heart, 1 paper Lung multiple lesions, 1 paper	
Pure lipoma	Broad ligament, 2 papers Round ligament, 1 paper	Ovarian thecoma (Vilallonga R. et al.) Cervical carcinoma (Dilek TU et al., 2006) Endometrial carcinoma (Di Gesu G. et al., 1998) Brest cancer (Uner A. et al., 2003) Struma ovarii (Dey P. et al., 1993) Benign Brenner tumor (Khatib Y. et al., 2015)

## Data Availability

The data that support the findings of this study are available from the corresponding author upon reasonable request.

## References

[1] Kurman R. J., Ellenson L. H., Ronnett B. M. (2019). *Blaustein’s Pathology of the Female Genital Tract*.

[2] Khatib Y., Patel R. D., Dande M. (2015). Uterine lipoma with a coincidental Brenners tumor in the ovary in postmenopausal women: a case report. *Journal of Midlife Health*.

[3] Ghosh B., McKeown B., Gumma A. (2011). Lipoleiomyoma. *Case Reports*.

[4] Vamseedhar A., Shivalingappa D. B., Suresh D. R., Geetha R. L. (2011). Primary pure uterine lipoma: a rare case report with review of literature. *Indian Journal of Cancer*.

[5] McDonald A. G., Dal Cin P., Ganguly A. (2011). Liposarcoma arising in uterine lipoleiomyoma. *The American Journal of Surgical Pathology*.

[6] Scurry J., Hack M. (1990). Leiomyosarcoma arising in a lipoleiomyoma. *Gynecologic Oncology*.

[7] Di Gesu G., Cormio G., Di Vagno G., Melilli G. A., Renzulli G., Selvaggi L. (1998). Pure lipoma of the uterus in association with endometrial carcinoma. *European Journal of Obstetrics & Gynecology and Reproductive Biology*.

[8] Vural C., Özen Ö., Demirhan B. (2011). Intravenous lipoleiomyomatosis of uterus with cardiac extension: a case report. *Pathology-Research and Practice*.

[9] Fukunaga M. (2003). Benign “metastasizing” lipoleiomyoma of the Uterus. *International Journal of Gynecological Pathology*.

[10] Seo B. F., Choi J. S., Shim H. S. (2018). Cutaneous intramuscular lipoma: a new subtype of intramuscular lipoma. *Indian Journal of Pathology and Microbiology*.

